# Purely Ligamentous Flexion-Distraction Injury in a Five-Year-Old Child Treated with Surgical Management

**DOI:** 10.7759/cureus.1130

**Published:** 2017-04-03

**Authors:** Ryan M Schiedo, William Lavelle, Nathaniel R Ordway, Tarush Rustagi, Mike H Sun

**Affiliations:** 1 Medical Student, Suny Upstate Medical University, Syracuse, NY; 2 Department of Orthopedic Surgery, Suny Upstate Medical University, Syracuse, NY

**Keywords:** chance fractures, flexion-distraction injury, pediatric, surgical management

## Abstract

Chance fractures by definition are a type of flexion-distraction injury with concomitant vertebral body fracture. Although uncommon in the pediatric population, they are associated with motor vehicle accidents and typically involve the thoraco-lumbar spine. Injury occurs when the spine rotates about a fixed axis, such as a lap belt. Our case reports the management of a five-year-old girl involved in a head-on collision who suffered a purely ligamentous flexion-distraction injury (Chance-type injury, without bone involvement) at the L2-L3 vertebral level. Previously these injuries were managed conservatively with serial casting; however, we present a case in which surgical management was used. A five-year-old girl sustained multiple injuries after being involved in a high-speed motor vehicle accident. At presentation, there was obvious abdominal bruising with a seat-belt sign and marked kyphosis of the spine with severe tenderness at the L2-L3 level. She required immediate exploratory laparotomy for her intraabdominal injuries. After stabilization, an orthopedic consult was deemed necessary. She was found to have occipital-cervical injury with mild anterolisthesis of C2 on C3 and disruption of the apical ligament. There was evidence of bilateral dislocation of the L2-L3 facet joints with marked disruption of the posterior ligaments and a hematoma sack. She required open reduction and internal fixation with an L2-L3 laminectomy, pedicle screw and rod placement. The kyphotic deformity was reduced using a compression device and stable alignment was achieved intraoperatively.

This was a rare and difficult case with limited evidence on the appropriate management of such an injury. Due to the severe instability of her injury, a surgical approach was taken. At two years postoperative, the patient is neurologically intact and pain free. Imaging revealed stable alignment of her lumbar hardware. Ultimately, this has resulted in an excellent outcome at the current follow-up.

## Introduction

Chance fractures by definition are a type of flexion-distraction injury with concomitant vertebral body fracture. Although uncommon in the pediatric population, they are associated with motor vehicle accidents and typically involve the thoraco-lumbar spine. The injury occurs when the spine rotates about a fixed axis, such as a lap belt, that is riding above the iliac crests during a collision [1]. Historically, Chance-type injuries have been treated with conservative management through casting or bracing in a hyper-extended position with concomitant bed rest [2-3]. In 1994, Greenwald and Mann published the first report detailing clinical outcomes of pediatric patients treated surgically through open reduction and internal fixation [4]. More recent reports have been published suggesting operative management of flexion-distraction injuries. In 2011, Arkader, et al. concluded that surgical treatment appears to provide better clinical outcomes from Chance fractures compared to conservative management [5].

Our case reports the management of a five-year-old girl, involved in a head-on collision, who suffered a purely ligamentous flexion-distraction injury (Chance-type injury, without bone involvement) at the L2-L3 vertebral level. Her treatment involved surgical reduction and two-level fusion of the injury, which ultimately resulted in complete recovery without recurrent pain or neurological deficit. We believe this case provides an anecdotal experience with an extremely rare injury and further illuminates Chance-type injury management. The patient’s guardian provided written informed consent for the publication of this article.

## Case presentation

A previously healthy five-year-old girl was involved in a high-speed head-on collision in a 55 mph speed zone. She was wearing a 3-point restraining seatbelt and sitting in the rear of the vehicle at the time of the accident. At the scene, paramedics found her to be conscious and alert. She was airlifted to the emergency room with full spinal precautions and wearing a hard cervical collar. She complained of severe abdominal pain and denied any numbness, paresthesias, or weakness in her legs. 

**Figure 1:**
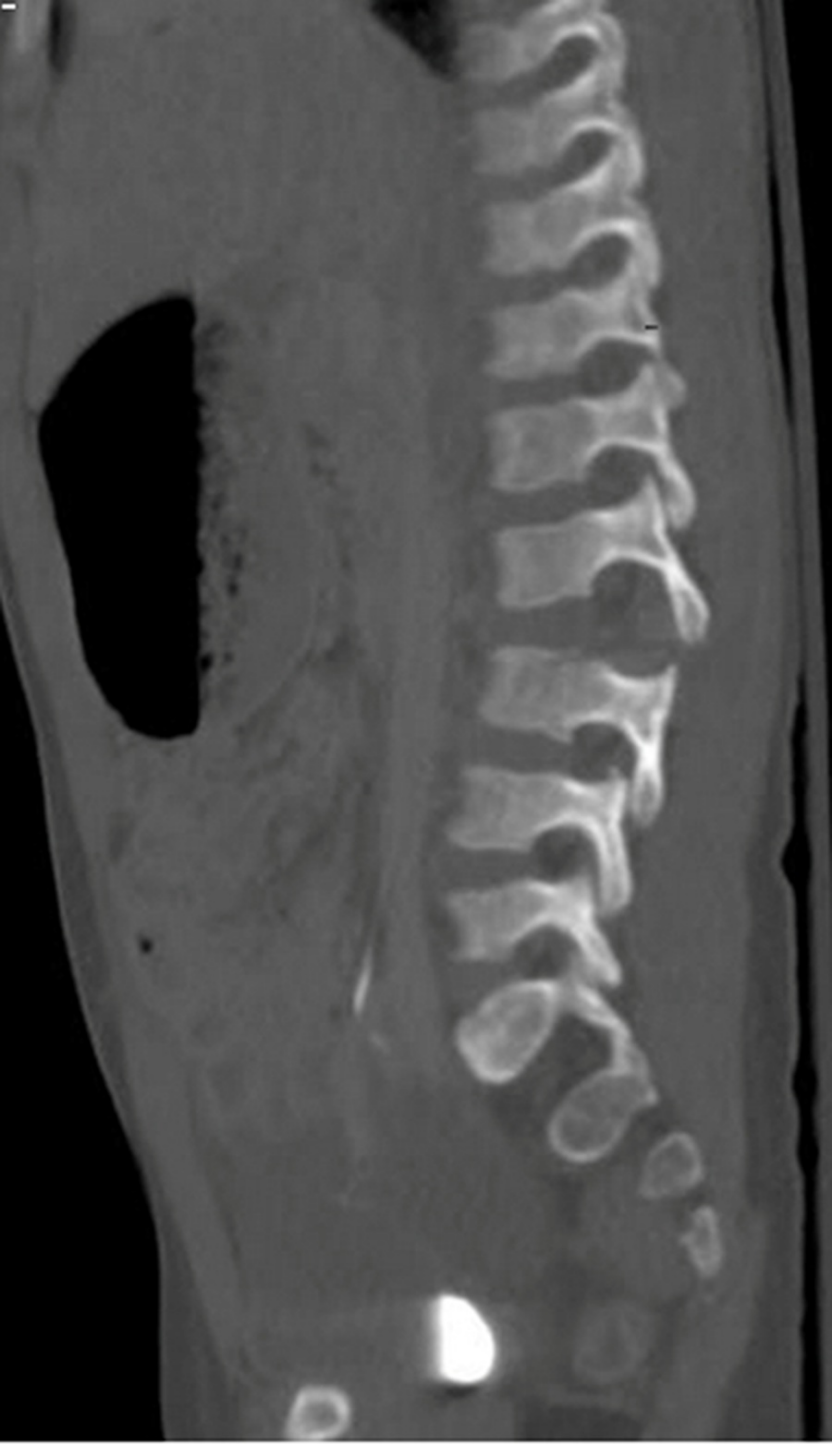
Sagittal CT scan with contrast of the lumbar spine right of midline. There is angulation at the L2-3 level with widening of the posterior intervertebral disc space and the spinous processes, findings consistent with ligamentous injury. Fracture was not detected in this series.

**Figure 2:**
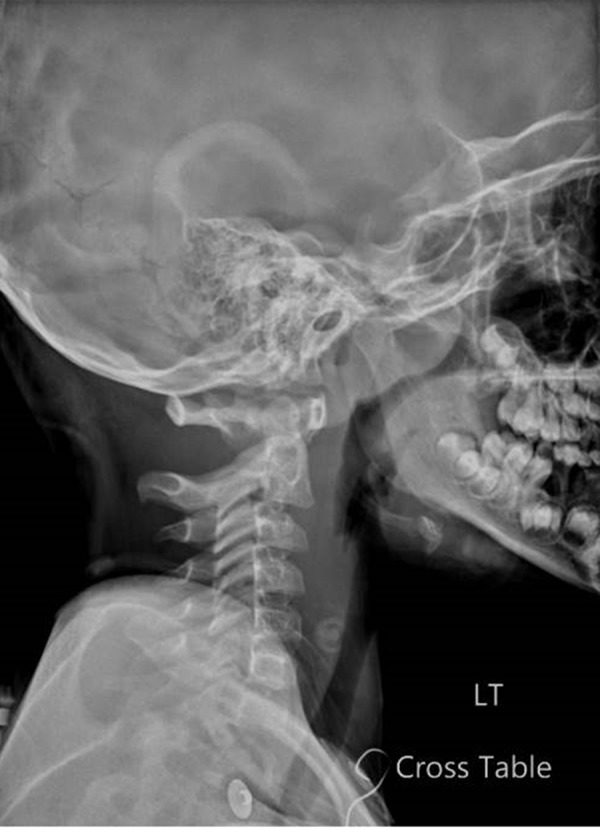
Lateral X-ray of the cervical spine revealing mild anterolisthesis of C2 on C3.

Physical examination found her to have a large abrasion on her right forehead, ecchymosis across her abdomen (seat-belt sign) with abrasions, swollen left lower leg, as well as tender and swollen lumbar spine with marked kyphosis. Focused assessment with sonography for trauma (FAST) and computed tomography (CT) revealed: a non-distended tender abdomen, intraperitoneal fluid, closed left distal tibia fracture, Chance-type flexion-distraction lumbar injury with a L2-3 perched facet (Figure 1) and anterolisthesis of C2 on C3 (Figure 2). Her CT was negative for intra-cranial bleeds or fractures.

Subsequently, she underwent an emergent exploratory laparotomy where she had small bowel perforations, cecal perforations and a deserosalized ileum. Small bowel resection, right hemicolectomy and an ileo-ileal anastomosis were performed to repair her enteric injuries. She tolerated the surgery well and was moved to the pediatric intensive care unit (PICU) where an orthopedic consult was requested for her spinal injuries.

**Figure 3:**
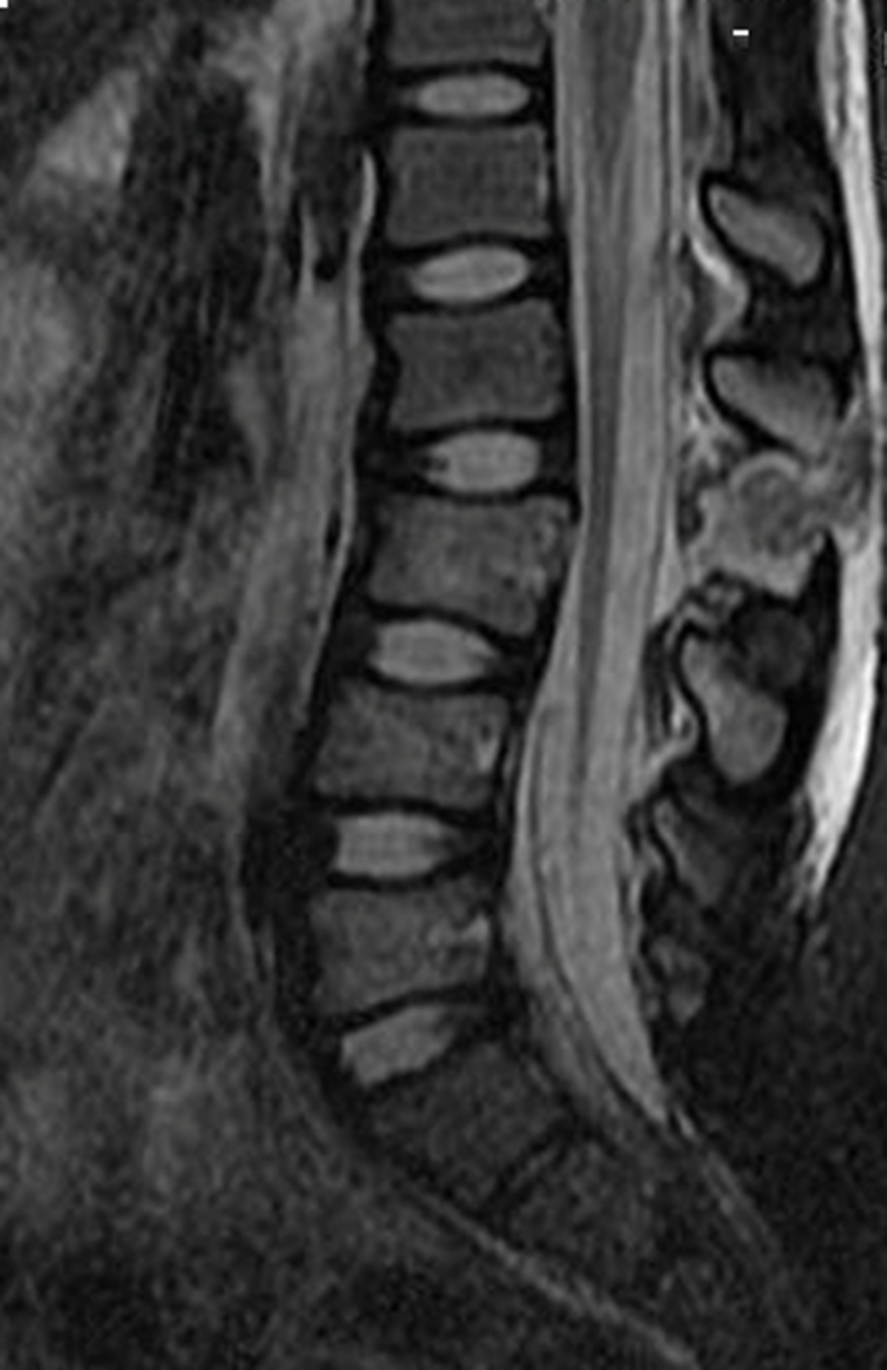
T2 weighted MRI of the lumbar spine without contrast revealing a hyperflexion injury with rupture of the interspinous ligaments and ligamentum flavum at the L2-L3. The increased signal intensity is consistent with the formation of a hematoma sac.

**Figure 4:**
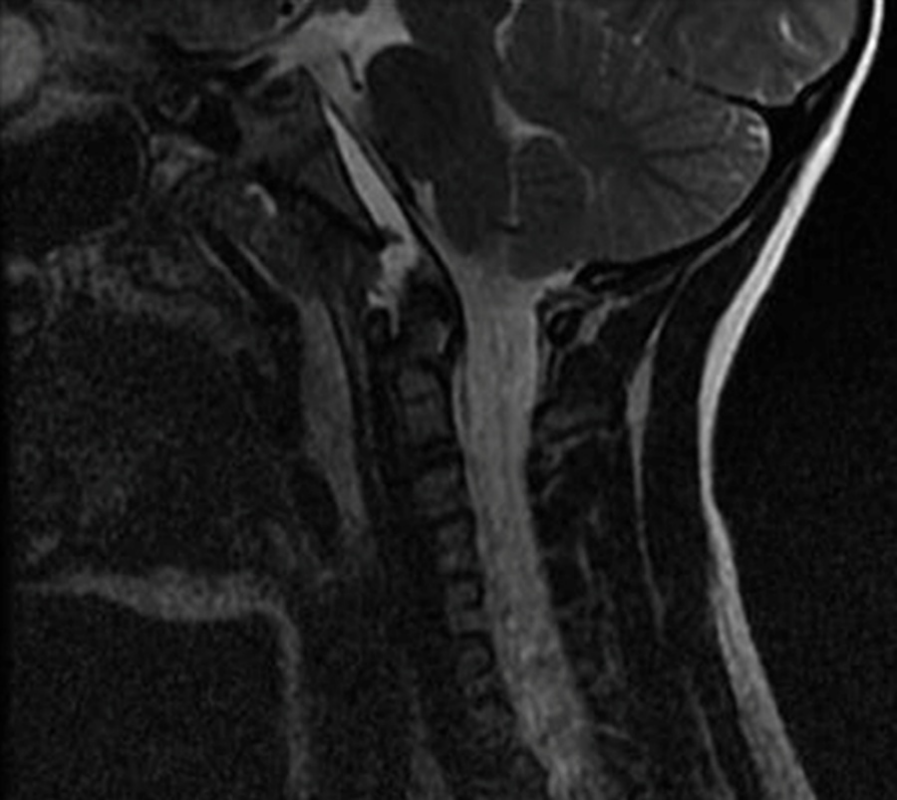
T2 weighted sagittal MRI of the cervical spine without contrast. There is hyperintesity underneath the tectorial membrane extending from C2 to the upper margin of the clivus with apparent discontinuity of the apical ligament.

Magnetic resonance imaging (MRI), taken after her abdominal surgery while sedated, confirmed hyperflexion injuries involving her lumbar and cervical spine (Figures 3-4). There was evidence of a bilateral dislocation of the L2-3 facet joints with marked disruption of the posterior ligamentous structures making it highly unstable. Her cervical MRI revealed flexion injury with no spinal cord signal changes consistent with injury. She had mild anterolithesis of C2 on C3 and disruption of the apical ligament. Due to the combination of a ligamentous injury with facet joint diastasis and severe kyphosis in her lumbar spine, we felt that operative management was warranted. Her cervical injury was considered stable and treated conservatively in a Miami J collar.

**Figure 5:**
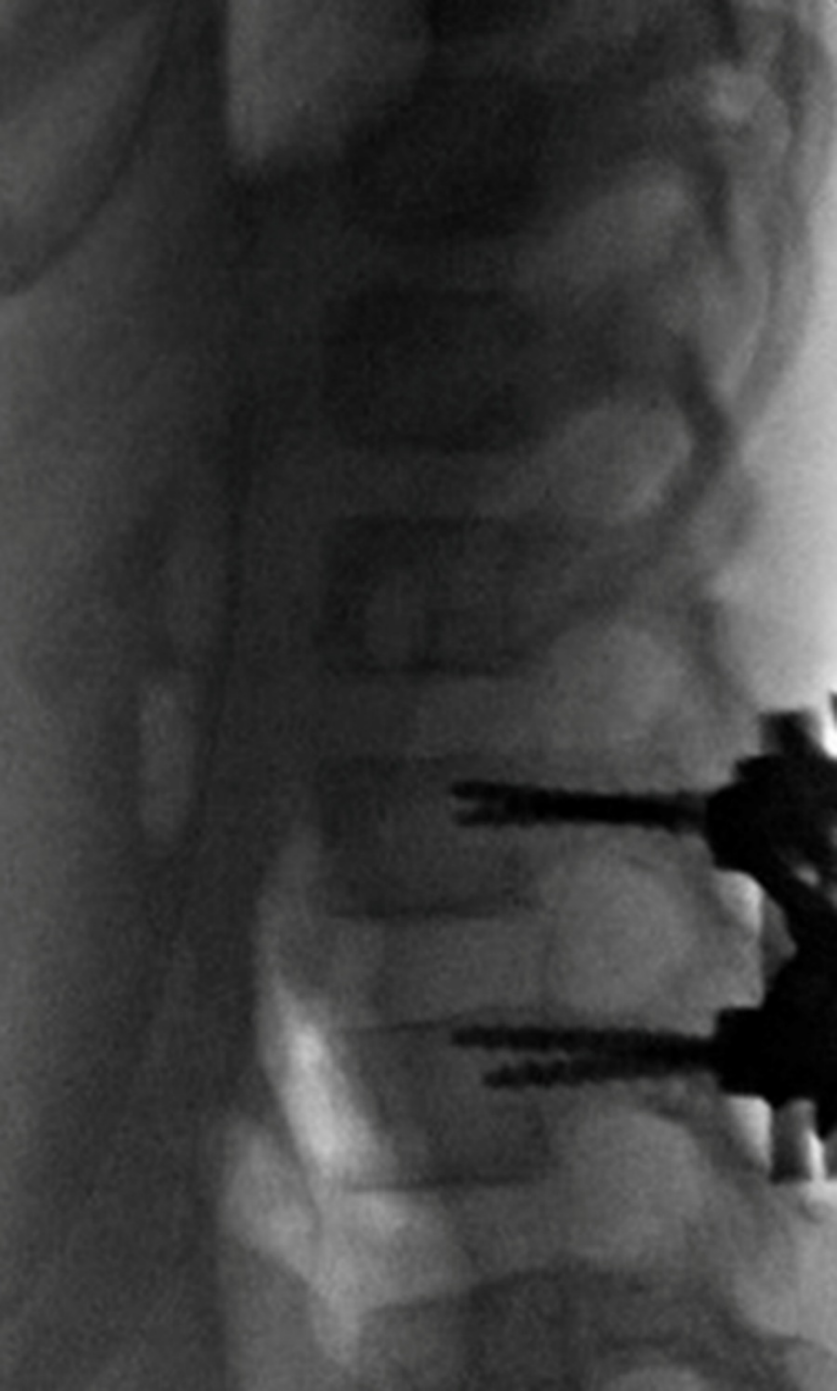
Intraoperative X-ray showing internal fixation and reduction of the facet joint at L2-L3.

Forty-eight hours after her abdominal procedure, she was considered medically stable to undergo surgical management of her lumbar injuries. Intraoperatively, the posterior ligamentous structures, including the ligamentum flavum, were found buckled and torn. The facet joints were dislocated with a hematoma noted. An L2-3 laminectomy was performed to decompress the dural sac, drain the hematoma and clear the facet joint capsule. Pedicle screws were placed bilaterally in L2 and L3. The dislocated facet joints were carefully reduced symmetrically using a compression device simultaneously on both sides. The kyphotic deformity was reduced and stable alignment achieved (Figure 5).

The patient remained in the hospital for 16 days. She was sent home in a pinless halo for the cervical spine injury. At her first follow-up, 26 days after her initial injury, the patient was independently ambulatory and pain free. The patient was last seen at a two year follow-up and is doing well. She is neurologically intact and reports no discomfort in her back or neck.

## Discussion

There is a paucity of information on the management of pediatric spine injuries due to their uncommon occurrence, which represents less than five percent of all spine injuries [6]. The most common cause of spinal injury in the pediatric population is motor vehicle crashes (MVC), and although seat-belts have been shown to reduce the morbidity and mortality associated with MVCs, improper restraint is the leading cause of flexion-distraction injuries [7]. If used according to the manufacturer’s recommendations, 3-point restraints can be effective at reducing the incidence of flexion-distraction injuries [1]. We presume our patient did not meet the manufacturer’s recommendations and suffered her injury due to her inherent anatomical differences from the recommended population. Children under the age of eight have an increased head to body ratio, as well as, underdeveloped iliac crests, and during rapid deceleration, the force of impact is distributed to their abdomen and lumbar spine instead of the pelvis [8]. In young children, the level of injury tends to occur around L3, a finding consistent with our case [9].

Flexion-distraction injuries have historically been managed conservatively with casting or bracing in a hyper-extended position. In 1994 Greenwald and Mann published the first report detailing the clinical outcome of lumbar flexion-distraction injuries managed with operative reduction and two-level fusion [4]. Since their findings and recommendation for operative reduction and fusion, more recent publications have outlined management of Chance-type injuries. In 2011, Arkader, et al. reviewed the treatment of 35 patients with Chance fractures and their management. They concluded surgical treatment appears to provide better clinical outcomes than the historical casting and bracing [5]. In 2013, Suttor, et al. concluded similar results stating operative management of pediatric chance injuries with instrumentation resulted in excellent clinical and radiological outcomes [10].

## Conclusions

Spinal injury in the pediatric population is a significant concern as appropriate treatment of these types of injuries is critical in order to prevent further neurological damage and deformity. The patient in our case was treated surgically with open reduction and internal fixation. She has been pain free and neurologically intact since her treatment and is continuing to develop normally.

## References

[1] Gordon ZL, Gillespie RJ, Ponsky TA (2009). Three siblings with chance fractures: the importance of 3-point restraints. J Pediatr Orthop.

[2] Glassman SD, Johnson JR, Holt RT (1992). Seatbelt injuries in children. J Trauma.

[3] Rumball K, Jarvis J (1992). Seat-belt injuries of the spine in young children. J Bone Joint Surg Br.

[4] Greenwald TA, Mann DC (1994). Pediatric seatbelt injuries: diagnosis and treatment of lumbar flexion-distraction injuries. Paraplegia.

[5] Arkader A, Warner WC Jr, Tolo VT (2011). Pediatric chance fractures: a multicenter perspective. J Pediatr Orthop.

[6] Goodwin CR, Recinos PF, Jallo GI (2012). Pediatric spinal trauma. Neurosurg Q.

[7] Knox JB, Schneider JE, Cage JM (2014). Spine trauma in very young children: a retrospective study of 206 patients presenting to a level 1 pediatric trauma center. J Pediatr Orthop.

[8] Louman-Gardiner K, Mulpuri K, Perdios A (2008). Pediatric lumbar chance fractures in British Columbia: chart review and analysis of the use of shoulder restraints in MVAs. Accid Anal Prev.

[9] Reilly CW (2007). Pediatric spine trauma. J Bone Joint Surg Am.

[10] Suttor S, Gray R, Bridge C (2013). Operative treatment of chance injuries in the paediatric population. Eur Spine J.

